# Biological Characteristics and Genetic Analysis of a Highly Pathogenic *Proteus Mirabilis* Strain Isolated From Dogs in China

**DOI:** 10.3389/fvets.2020.00589

**Published:** 2020-10-07

**Authors:** Renge Hu, Xue Wang, Inam Muhamamd, Yiming Wang, Wenlong Dong, Haipeng Zhang, Yu Wang, Shuming Liu, Yunhang Gao, Lingcong Kong, Hongxia Ma

**Affiliations:** ^1^College of Animal Science and Technology, Jilin Agricultural University, Changchun, China; ^2^The Key Laboratory of Animal Production, Product Quality and Security, Ministry of Education, Jilin Agricultural University, Changchun, China

**Keywords:** *Proteus mirabilis*, antibiotic resistance, complete genome sequencing, resistance gene, virulence gene

## Abstract

To evaluate the antimicrobial resistance and virulence gene characteristics of highly pathogenic *Proteus mirabilis*. In this study, we isolated *P. mirabilis* CC15031 from diarrhea dogs in China, tested the median lethal dose (LD_50_), and measured the minimum inhibitory concentration (MIC) of 10 different antibiotics commonly used in veterinary clinic. Meanwhile, we presented the complete genome sequence annotations to analyze the virulence and resistance formation mechanism. The results showed that the CC15031 presented relatively potent pathogenicity in mice (LD_50_ = 0.57 × 10^6^ CFU) and exhibited a high degree of resistance to all the tested antimicrobial agents. The CC15031 genome of 4,031,742 bp with 3,745 predicted genes had an average gene length of 917 bp and 38.99% guanine-cytosine content. A new variant of an integrative and conjugative element with a type IV secretion system (217,446 bp) conferring multidrug resistance was identified and characterized by structural analysis in CC15031. These data provide a foundation for understanding the genomic features and antimicrobial resistance mechanisms of this pathogen.

## Introduction

*Proteus mirabilis* (*P. mirabilis*) has been recognized as a significant zoonotic pathogen, causing a variety of diseases, including diarrhea, urinary tract infections, and keratitis and considered the second most common zoonotic bacterium after enteropathogenic *Escherichia coli* in human medicine (1). In recent years, it has been considered to be a repository for virulence and resistance genes and has become a potential public health concern. Previous studies indicated that severe drug resistance had been developed in *P. mirabilis* against commonly used antibiotics. Thus, the emergence of drug-resistant strains has posed clinical difficulties and become a potential threat to public health (2).

Generally, *P. mirabilis* accounts for about 90% of *Proteus* infections and is considered a community-acquired infection (3). Virulence determinants acquired by *P. mirabilis* induce infections successfully (4). Drug resistance genes and drug resistance gene islands, such as the extended-spectrum β-lactamase gene (*bla*_*VEB*−6_), have been found in *P. mirabilis* in many countries (5, 6). Also, the acquired quinolone resistance gene (*qnrA1*) was isolated from humans and animals in France (6). Recently, two novel *Salmonella* genomic island 1 (SGI1) variants, SGI1-PmBC1123, and SGI1-PmSC1111, which carried multiple drug resistance genes, were described in *P. mirabilis* strains isolated from Food Animals in China (7). Also, some studies used PCR sequencing and demonstrated that *P. mirabilis* carries variant virulence islands (8, 9).

Since 2015–2017, we have found that the diarrhea disease of dogs caused by *P. mirabilis* in the Changchun area of China is relatively severe, but the underlying antimicrobial resistance and pathogenicity are yet unknown. Although the genomes of many human-sourced *P. mirabilis* (for example, NCTC1178, 81–176) have been sequenced, to date, *P. mirabilis* with multidrug resistance and virulence in dogs have not been sequenced and reported. In this study, one strain of *P. mirabilis* with high pathogenicity and drug resistance, which was isolated, was selected and analyzed by whole-genome sequence, and a new SXT/R391 ICE variant was found.

## Materials and Methods

### Sample Collection and Bacterial Isolation

The samples were collected from diarrheal specimens of 62 dogs suffering from bacterial enteritis during 2015–2017. The samples were inoculated on lysogeny broth (LB) agar medium and brain heart infusion (BHI) agar medium and cultured at 37 °C for 12 h. The bacterial growth was observed, and the colonies with the same morphology and migration ability were selected for streak culture to obtain the pure culture strain. Then, the genomes of the isolates were extracted, amplified, and sequenced by PCR using *16S* ribosomal RNA (rRNA) primers. The sequencing results were subjected to BLAST analysis at the National Center for Biotechnology Information website. Strains with 99% sequence homology to the *P. mirabilis* 16S rRNA gene were selected for further analysis.

### Median Lethal Dose Determination

The median lethal dose (LD_50_) of *P. mirabilis* was determined by the modified Kirschner method, and the colony count of each strain was carried out (10). Then, three dose groups of 1 × 10^7^-1 × 10^9^ CFU/ml were set through the pre-experiment. Eight mice, an equal number of males and females, were randomly selected from each group. Each dose group was injected intraperitoneally with a 0.2 ml bacterial solution at different gradient concentration. The mice in each group were kept in isolation, the death rate was observed for 3 days, and the LD_50_ was calculated (11). The data were statistically analyzed using SPSS (19.0) software. All the methods were carried out by the National Institutes of Health guidelines and protocols for laboratory animal use and proper care, approved by the Animal Care and Use Committee of Jilin Agricultural University.

### Antimicrobial Susceptibility Assay

The antimicrobial sensitivity phenotypes of *P. mirabilis* were determined by the broth dilution method using Muller–Hinton (MH) agar plates following Clinical and Laboratory Standards Institute guidelines M100-26. The resistance breakpoint was as follows: enrofloxacin > 2 μg/ml, clindamycin > 4 μg/ml, ceftriaxone > 4 μg/ml, sulfamethoxazole > 512 μg/ml, gentamicin > 16 μg/ml, chloramphenicol > 32 μg/ml, florfenicol > 16 μg/ml, doxycycline > 16 μg/ml, ciprofloxacin > 4 μg/ml, and erythromycin > 32 μg/ml. *E. coli* ATCC25922 strain was used for quality control.

### Whole-Genome Sequencing

The total DNA of *P. mirabilis* CC15031 was extracted using a genomic DNA kit (A&A Biotechnology, Gdansk, Poland). Then, the SMRT Bell library was constructed. The purified fragments were solubilized in the buffer, and the specific size fragments were screened with Blue Pipin and purified with AMpure PB magnetic beads. The library was constructed quantitatively based on Qubit estimation, and the insert size was detected with Agilent 2100. Then, the fragments were sequenced on the PacBio platform. The sequencing data were deposited in GenBank, with a CP031532 accession number.

### Bioinformatics

The gene function was subsequently annotated with GO (Gene Ontology), KEGG (Kyoto Encyclopedia of Genes and Genomes), COG (Cluster of Orthologous Groups of proteins), and NR (Non-Redundant Protein Database). ARG (antibiotic resistance gene) analysis was performed using the Resistance Gene Identifier (v4.1.0) tool of the CARD (The Comprehensive Antibiotic Resistance Database), CGE ResFinder 3.1, and IslandPath-DIOMB software (version 0.2) was used to predict the gene islands by detecting the dinucleotide biases and mobility of genes to identify the gene islands and the potential horizontal gene transfers.

## Results

### Minimum Inhibitory Concentration and Median Lethal Dose of *Proteus mirabilis* Isolates

One *P. mirabilis* strain was selected from diarrheal specimens of dogs and identified by the 16S rRNA sequencing. Antibiotic resistance and pathogens of the isolates were evaluated by the median lethal dose (LD_50_) and the minimum inhibitory concentration (MIC) test (Tables S1, S2). The LD_50_ of the bacterial strain *P. mirabilis* CC15031 isolated in this study was determined by the modified Kirschner method and showed relatively strong virulence to mice (LD_50_ = 0.57 × 10^6^CFU). Following standardized international definitions, the MICs of different antimicrobial agents against the *P. mirabilis* CC15031 showed a high-level antibiotic resistance, representing a multidrug-resistant strain (Table 1).

**Table 1:** MICs (microgram per milliliter) of different antibiotics agains *P. mirabilis* CC15031.

**Strain no**.	**ENR**	**CLI**	**SUL**	**FOR**	**CEF**	**DOX**	**CIP**	**GEN**	**ERY**	**CHL**
CC15031	16	>512	>512	32	>512	128	16	256	>512	256

*ENR, Enrofloxacin; CLI, Clindamycin; SUL, Sulfamethoxazole; FOR, Florfenicol; CEF, Ceftriaxone; DOX, Doxycycline; CIP, Ciprofloxacin; GEN, Gentamicin; ERY, Erythromycin; CHL, Chloramphenicol677*.

### Whole-Genome Features of *P. mirabilis* CC15031

Based on the above LD_50_ and MICs studies, CC15031 was selected for whole-genome sequencing to elucidate potential mechanisms underlying the multidrug resistance and high virulence, a Circos circular representation of the CC15031 genome with annotated genes was constructed (Figure S2). The CC15031 genome is comprised of one chromosome and a plasmid. It is 4,031,742 bp long, containing 3,745 predicted genes. The average length of each gene was 917 bp. Guanine-cytosine content of this genome was 38.99%, which was similar to other reported *P. mirabilis* genomes. The complete nucleotide sequence of CC15031 characterized in this study was submitted to GenBank and assigned accession number CP048787.

### Virulence-Associated Genes in *Proteus mirabilis* CC15031

The results of comparing the identity values ≥ 80% were statistically analyzed based on the Virulence Factor Database. A large number of virulence-associated genes were predicted in the genome of CC15031 (Table S3). Among the selected genes involved in the capsular polysaccharide, elongation factor Tu (EF-Tu) and adhesion were identified. In the biofilm formation system, the genome of CC15031 contained 26 virulence factors, which involved 44 genes (Table 2). As for motility characteristics, 9 different diverse flagellum virulence factors related to 51 genes and 12 fimbriae virulence factors related to 47 genes were identified in the CC15031 genome (Tables 3, 4). Among all the obtained virulence factors, two exogenous adhesive molecules, AI396 (*M. catarrhalis*) and CVF228 (*Listeria*), and three exogenous pilus virulence factors, AI102, CVF825 (*Hemorrhagic coli* pili), and CVF6259 (*E. coli* common pilus) were identified. Genes associated with flagella formation *flaA, flgD* were observed in CC15031 genome and no *flaB* found. Compared with the genes of other *P. mirabilis*, CC15031 contained several typical secretion systems (TNSSs), including one T1SS, six T3SS, and nine T6SS. A total of 15 gene islands with a total length of 248,432 bp and an average length of 16,562 bp of each gene were predicted using IslandPath-DIOMB software (version 0.2) (Figure S3).

**Table 2:** Biofilm formation-related gene annotations of CC15031 based on the Virulence Factor Database.

**No**.	**VF_id**	**VF_name**	**Related_genes**
1	AI072	*Proteus*-like (MR/P) fimbriae, mannose resistant (biofilm formation)	*mrpA, mrpB, mrpC, mrpD, mrpE, mrpF, mrpG, mrpH, mrpI, mrpJ*
2	AI330	OmpA	*ompA*
3	AI331	NlpI	*ECS88_3547*
4	AI392	MOMP	*CT396*
5	AI396	*M. catarrhalis* adherence protein (McaP)	*gbpA*
6	CVF010	PhoPQ	*phoP, phoQ*
7	CVF043	O-antigen	*YPK_3179, YE105_C0173, wzz*
8	CVF228	*Listeria* adhesion protein	*lap*
9	CVF335	(p)ppGpp synthesis and hydrolysis	*relA*
10	CVF349	Mip	*mip*
11	CVF380	LPS	*bplF*
12	CVF381	LPS-modifying enzyme	*pagP*
13	CVF383	LPS	*acpXL, fabZ*
14	CVF495	Exopolysaccharide	*mrsA/glmM, pgi*
15	CVF523	Alginate regulation	*algW, mucD, mucP*
16	CVF567	Polysaccharide capsule	*BCE_5384*
17	CVF651	Trehalose-recycling ABC transporter	*sugC*
18	CVF758	FarAB	*farA, farB*
19	CVF759	MtrCDE	*mtrC, mtrD*
20	CVF799	OmpA	*ompA*
21	CVF834	LPS	*glmU*
22	VF0056	LPS	*kdtB*
23	VF0091	Alginate	*algU*
24	VF0260	RelA	*relA*
25	VF0444	Lap	*lap*
26	VF0463	BfmRS	*bfmR*

*MR/P, mannose-resistant/Proteus-like*.

**Table 3:** Flagellum-related gene annotation of CC15031.

**No**.	**VF_id**	**VF_name**	**Related_genes**
1	AI139	Peritrichous flagella	*flgI, cheB, tar/cheM, fliS, fliN, fliO*
2	AI142	Lateral flagella	*lafK*
3	AI145	Peritrichous flagella	*cheA, cheD, cheR, cheW, cheY, cheZ, motA, motB*
4	AI149	Polar flagella	*fleR/flrC*
5	CVF039	Flagella (cluster I)	*flgC, flgE, flgF, flgG, flgK, flgL, flgM, flgN, flhA, flhB, flhC, fliE, fliF, fliI, fliL, fliP, fliR*
6	CVF382	Flagella	*flaA, flgD*
7	CVF643	Flagella	*tsr*
8	VF0394	Flagella	*flgB, flgH, flgJ, flhD, fliA, fliD, fliG, fliH, fliJ, fliM, fliQ, fliT, fliZ*
9	VF0473	Polar flagella	*nueA, flmH*

**Table 4:** Fimbriae-related gene annotation of CC15031.

**No**.	**VF_id**	**VF_name**	**Related_genes**
1	AI042	F18 fimbriae	*fedC*
2	AI046	Sfp fimbriae	*sfpC, sfpD*
3	AI070	PMF pili	*pmfA, pmfC, pmfD, pmfE, pmfF*
4	AI071	FIMBRIAL operon regulator	*ucaA, PMI0532, PMI0533, PMI0534, PMI0535*
5	AI072	*Proteus*-like (MR/P) fimbriae, mannose resistant (biofilm formation)	*mrpA, mrpB, mrpC, mrpD, mrpE, mrpF, mrpG, mrpH, mrpI, mrpJ*
6	AI075	TYPE 1 fimbriae	*fimA, fimF*
7	AI078	Pix pilus	*pixD, pixA*
8	AI083	Type 1 fimbriae	*fimC*
9	AI090	F9 fimbriae	*fimH*
10	AI097	Type IV pili	*rpoN, vfr*
11	AI102	*Hemorrhagic Coli* pili (HCP)	*hofB*
12	AI117	Type IV pili	*pilT*
13	CVF003	Fim	*fimA*
14	CVF425	P fimbriae	*papF*
15	CVF426	Type I fimbriae	*fimC, fimD, fimF, fimG*
16	CVF486	Type IV pili	*comE/pilQ*
17	CVF518	Type IV pili biosynthesis	*pilR*
18	CVF625	*E. coli* common pilus (ECP)	*ecpA, ecpB*
19	CVF825	*Hemorrhagic E.coli* pilus (HCP)	*ppdD*
20	VF0082	Type IV pili	*pilR*
21	VF0222	S FIMBRIAE	*sfaE*
22	VF0401	Type IV pili	*pilL*

*PMF, P. mirabilis fimbriae; MR/P, mannose-resistant/Proteus-like*.

### Resistance Determinants in *Proteus mirabilis* CC15031

The genome sequencing data showed that CC15031 contained 18 resistance genes (*catA4, tetJ, ant(2*″*)-Ia, ereA, ble, sul1, catB, blaOXA-1, AAC(6*′*)-Ib, aac(3)IV, SJL2, cml_e3, blaCTX-M, aph(3*′*)-Ia, tetC, aph(3*′*)-Ia, ant(3*″*)-Ia*, and *dfrA1*). These genes may confer resistance to penicillins, cephalosporins, macrolides, aminoglycosides, tetracyclines, and sulfonamides in CC15031 (Table 5).

**Table 5:** Drug resistance gene statistics of chromosome.

**Gene ID**	**Resistance type**	**Min identity**	**Antibiotic resistance**	**Identity**
GM001349	*catA4*	95	Chloramphenicol	98.78
GM003010	*tetJ*	87	Tetracycline	98.91%
GM003066	*ant (2)Ia*	90	Tobramycin, kanamycin, sisomicin, dibekacin, gentamicin	100%
GM003067	*ereA*	80	Erythromycin	100%
GM003076	*ble*	95	Bleomycin	100%
GM003080	*sulI*	80	Sulfonamide	100%
GM003083^a^	*catB3*	85	Chloramphenicol	100%
GM003084^a^	*bla_*OXA*−1_*	80	Cloxacillin, penicillin	100%
GM003085^a^	*aac(6′)-Ib*	85	Isepamicin, netilmicin, tobramycin, amikacin, sisomicin, dibekacin	99.87%
GM003088^a^	*aac(3(6′)) Ib*	80	Netilmicin, tobramycin, sisomicin, dibekacin, gentamicin, apramycin	99.89%
GM003098^a^	*sulII*	80	Sulfonamide	100%
GM003102^a^	*cml-E3*	90	Chloramphenicol	100%
GM003117	*bla_*CTX*−*M*_*	75	Monobactam, penicillin, cephalosporin_iii, ceftazidime, cephalosporin_ii, cephalosporin_i	100%
GM003123	*Aph(3′) Ia*	80	Paromomycin, neomycin, kanamycin, lividomycin, ribostamycin, gentamincin_b	100%
GM003128	*tetC*	85	Tetracycline	99.66%
GM003181	*Aph(3′) Ia*	80	Paromomycin, neomycin, kanamycin, lividomycin, ribostamycin, centenarian	100%
GM003681	*aac(3′) Ib-cr*	90	Spectinomycin, streptomycin	100%
GM003683	*dfrA1*	95	Trimethoprim	100%

a *these genes are located on the new ICE with T4SS*.

### Characterization of a Multidrug-Resistant Genomic Element

**Figure 1:**
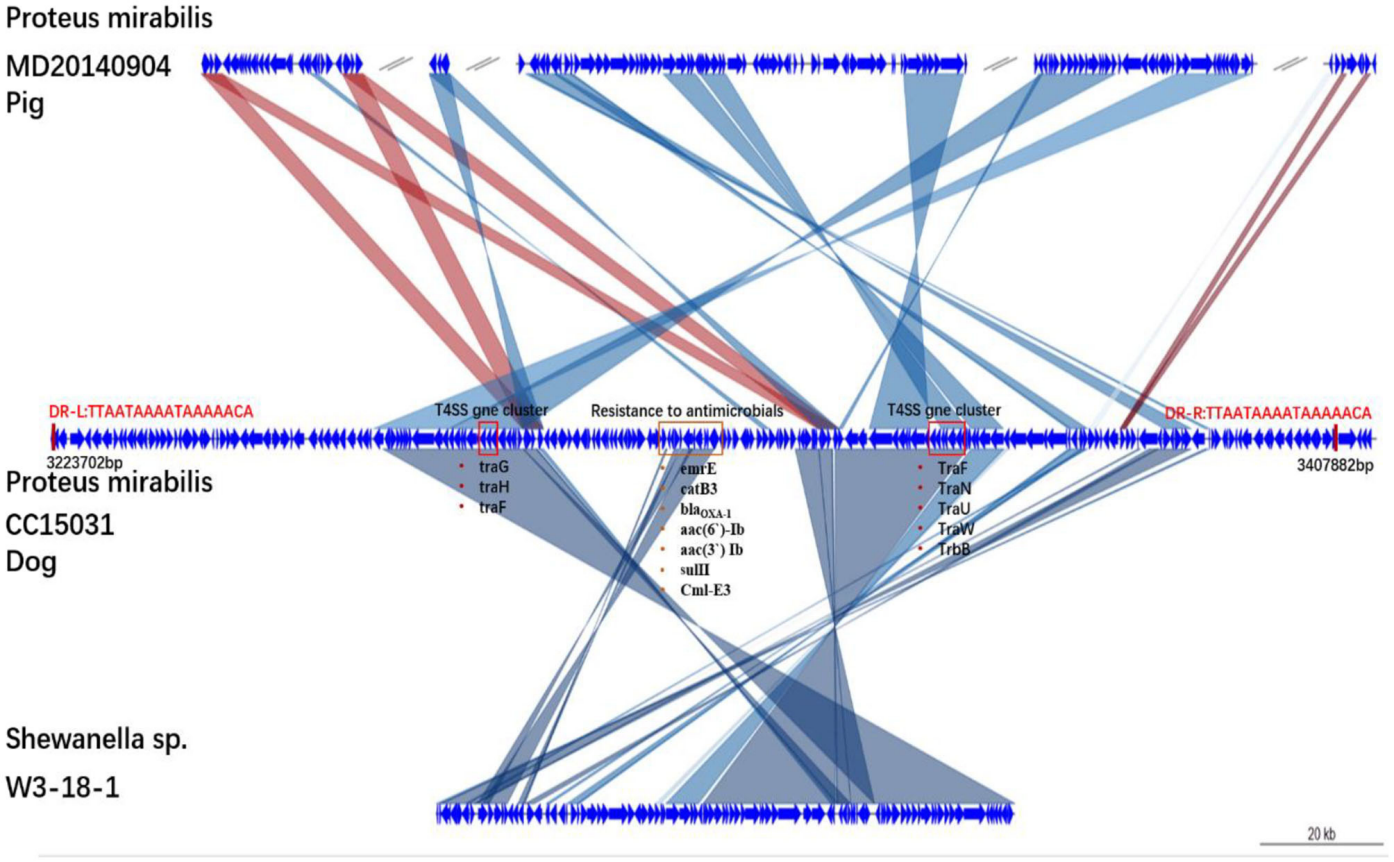
Genetic structures of SGI1-CC15031. Structures are drawn to scale from GenBank accession numbers MD20140904, W3-18-1. Genes and open reading frames are shown as arrows, and their orientations of transcription are indicated by the arrowheads. Shared regions with above 99% identity are indicated by shading. Resistance genes and integrase genes are in a red box. DR-L and DR-R represent the 18-bp direct repeats at the ends of SGI1.

Analysis of the chromosomal DNA of CC15031 revealed the presence of an integrative and conjugative element (ICE) region (217,446 bp in size with a guanine-cytosine content of 45%). The ICE flanked by a 17-bp target site duplication (TTAATAAAATAAAAACA). The ICE contained virulence factors, such as a T4SS, antibiotic resistance genes, such as the catB3 (encoding resistance to chloramphenicol), narrow-spectrum β-lactamase genes *bla*_*OXA*−1_ and *bla*_*CTX*−*M*_(encoding resistance to β-lactamase), *catB3* (encoding resistance to chloramphenicol), *aac(6*′*)-Ib*, and *aac(3*′*) Ib* (encoding resistance to aminoglycoside), *Cml-E3* (encoding resistance to chloramphenicol), and *sulII* (encoding resistance to sulfonamide) (Figure 1). The structural analysis showed that the ICE is a new ICE variant with T4SS. BLAST analysis showed that the new ICE had 70% nucleotide identity to *P. mirabilis* (MD20140904) and 56% nucleotide identity to *Shewanella* sp. (W3-18-1). Structures are drawn to scale from GenBank accession numbers. Shared regions with 99% identity are indicated by shading. Based on the comparative analysis, the ICE possessed many homologous genes in the gene cluster (link with dark line). Homologous regions include a series of DNA replication-related genes.

## Discussion

*P. mirabilis* is a conditioned zoonotic pathogen. It has been isolated from a variety of animal species, such as chicken, ducks, dogs, foxes, goats, mink, and other animals, and considered to be the host of storing drug resistance and virulence genes. However, the characteristics of drug resistance and virulence genes of the typical strains of *P. mirabilis* isolated from dogs have not yet been reported. A total of 12 *P. mirabilis* strains were isolated from the diarrheal specimen of dogs in Changchun during 2015–2017. From which, the *P. mirabilis* CC15031 is closely related to RCAD120210 isolated from geese in Sichuan, China. The results of the virulence and drug sensitivity test showed CC15031 possessed strong pathogenicity and high multidrug resistance.

The result of the drug resistance spectrum of *P. mirabilis* CC15031 was slightly different from that of other animal and human sources. In another study from Africa, 73 strains of *P. mirabilis* isolated from a University hospital were found to be susceptible to ampicillin, chlorobenzicillin, amoxicillin, tetracycline, compound triazole, erythromycin, and chloramphenicol. Also, high resistance to ampicillin, chlorobenzicillin, amoxicillin, tetracycline, compound triazole, and erythromycin was observed. Furthermore, *P. mirabilis* has developed drug resistance in recent years, which might be related to the excessive use of antibiotics by clinicians. Also, the specific physiological characteristics of the bacterium promoted its drug resistance.

The improvement in the genomic data can provide a reference for the typing and diagnosis of pathogenic microorganisms (12). A highly resistant and pathogenic strain *P. mirabilis* CC15031 was isolated in this study; however, the gene–environment related to drug resistance and pathogenicity of CC15031 is not yet clarified. Therefore, we sequenced the whole genome of CC15031 and analyzed it by comparative genomics, obtained the genome map, analyzed its gene function, and screened the drug resistance and virulence genes. CC15031 had a 4,031,742-bp long genome, consisting of a total of 3,705 coding genes, including 363 virulence factors and 18 drug resistance genes.

The particular feature of *P. mirabilis* was the excellent bacterial motility; so far, it is the microorganism with the highest amount of potential fimbriae. The biofilm, pili, and flagella of *P. mirabilis* play an important role in bacterial movement, adhesion and colonization, host invasion capability, and so on. As for the virulence factors, 26 virulence factors related to biofilm formation, 9 virulence factors related to flagellum formation, and 12 virulence factors related to pili formation were identified by using the Virulence Factor Database.

The three most studied *P. mirabilis* fimbriae were identified in CC15031, *P. mirabilis* fimbriae (AI070), uroepithelial cell adhesion (AI071), and mannose-resistant/*Proteus*-like (AI072). The fimbriae with the most genes annotated were the mannose-resistant/*Proteus*-like fimbriae (AI072). It is the major contributor to urinary tract infection, and the most well-studied fimbriae encoded by *P. mirabilis* could agglutinate erythrocytes independent of D-mannose (13). Our strain presents two additional copies of *ucaA* and other fimbriae related-protein genes that are not present in HI4320.

Also, the detection of *E. coli* (CVF6259) and *Hemorrhagic coli* (AI102, CVF825) pilus indicated the integration of exogenous virulence factors; this might be part of the reason for the increased virulence of the CC15031.

More importantly, we have discovered a new ICE variant with T4SS. Based on the comparative analysis, the ICE possessed many homologous genes in the gene cluster (link with dark line). Homologous regions include a series of DNA replication-related genes. Previous studies have found that a large number of ICEs have been identified in several genera of Gammaproteobacteria (http://db-mml.sjtu.edu.cn/ICEberg/) (14); most of them carry a large number of drug-resistant genes. In recent years, many ICEs had been reported in *P. mirabilis* (7, 15–18); most of them carry SXT/R391 (19). Notably, two SXT/R391 ICEs, ICEPmiJpn1 and ICEPmiSpn1 (20, 21), carry the *AmpC*-lactamase gene *blaCMY-2* mediating resistance to a broad spectrum of β-lactams and β-lactamase inhibitors, indicating that SXT/R391 ICEs can mediate the dissemination of clinically important resistance genes. Here, we characterized another novel ICE with T4SS that contained eight different antimicrobial resistance genes in *P. mirabilis*, including *blaOXA1* and *blaCTX-M*, which could confer resistance to penicillin and ceftazidime, respectively. This new ICE has a longer sequence than previously reported. Whether the four-type secretory system it carries mediates the more serious pathogenicity of the bacterium needs further research.

In conclusion, we found that *P. mirabilis* with explosive growth characteristics is the host of a large number of drug resistance and virulence genes, which might pose a threat to public health safety, requiring high attention. The biological and genetic evolutionary characteristics of *P. mirabilis* CC15031 will provide a reference for the prevention of human and animal diseases caused by *P. mirabilis*.

## Data Availability Statement

The datasets generated for this study can be found in the NCBI Database [accession: PRJNA596082].

## Ethics Statement

The animal study was reviewed and approved by the Animal Care and Use Committee of the Jilin Agricultural University.

## Author Contributions

LK, RH, and XW performed the experiments. RH, IM, YiW, WD, HZ, YuW, and SL collected the samples. RH and LK wrote the manuscript. HM, YG, and LK approved the final manuscript.

## Conflict of Interest

The authors declare that the research was conducted in the absence of any commercial or financial relationships that could be construed as a potential conflict of interest.
