# Supplementary material for: Biological Characteristics and Genetic Analysis of a Highly Pathogenic Proteus Mirabilis Strain Isolated From Dogs in China
Source: Front Vet Sci. 2020 Oct 7;7:589. doi: 10.3389/fvets.2020.00589 (PMC7575763; doi:10.3389/fvets.2020.00589)
Supplement: Supplementary file 1 [file Data_Sheet_1.docx]

Supplement Table S1. Determination of LD_50_ of 12 *P. mirabilis* isolates

| Strain No. | Bacterial concentration (CFU/mL) | Total number of mice | Dead mice number | LD_50_ |
| --- | --- | --- | --- | --- |
|  | 3.5×10^9^ | 8 | 6 |  |
| CC15042 | 3.5×10^8^ | 8 | 3 | 1.78×10^8^ |
|  | 3.5×10^7^ | 8 | 0 |  |
|  | 2.17×10^9^ | 8 | 6 |  |
| CC16101 | 2.17×10^8^ | 8 | 7 | 1.54×10^9^ |
|  | 2.17×10^7^ | 8 | 3 |  |
|  | 2.94×10^9^ | 8 | 8 |  |
| CC16012 | 2.94×10^8^ | 8 | 3 | 0.76×10^8^ |
|  | 2.94×10^7^ | 8 | 0 |  |
|  | 2.72×10^9^ | 8 | 7 |  |
| CC16071 | 2.72×10^8^ | 8 | 2 | 1.30×10^8^ |
|  | 2.72×10^7^ | 8 | 0 |  |
|  | 2.74×10^9^ | 8 | 8 |  |
| CC16091 | 2.74×10^8^ | 8 | 5 | 0.48×10^8^ |
|  | 2.74×10^7^ | 8 | 0 |  |
|  | 2.67×10^9^ | 8 | 7 |  |
| CC17011 | 2.67×10^8^ | 8 | 2 | 1.28×10^8^ |
|  | 2.67×10^7^ | 8 | 0 |  |
|  | 1.94×10^9^ | 8 | 5 |  |
| CC16082 | 1.94×10^9^ | 8 | 5 | 4.65×10^8^ |
|  | 1.94×10^9^ | 8 | 1 |  |
|  | 2.24×10^9^ | 8 | 6 |  |
| CC15071 | 2.24×10^8^ | 8 | 6 | 0.51×10^8^ |
|  | 2.24×10^7^ | 8 | 0 |  |
|  | 2.78×10^9^ | 8 | 7 |  |
| CC15091 | 2.78×10^8^ | 8 | 4 | 0.8×10^8^ |
|  | 2.78×10^7^ | 8 | 0 |  |
|  | 2.84×10^9^ | 8 | 8 |  |
| CC16092 | 2.84×10^8^ | 8 | 4 | 2.84×10^8^ |
|  | 2.84×10^7^ | 8 | 0 |  |
|  | 2.18×10^9^ | 8 | 8 |  |
| CC15031 | 2.18×10^8^ | 8 | 5 | 0.57×10^6^ |
|  | 2.18×10^7^ | 8 | 2 |  |
|  | 2.52×10^9^ | 8 | 8 |  |
| CC16051 | 2.52×10^8^ | 8 | 3 | 0.50×10^8^ |
|  | 2.52×10^7^ | 8 | 0 |  |

Supplement Table S2. MICs (μg/mL) of different agents against twelve *P. mirabilis* isolates

| Strain No. | ENR | CLI | SUL | FOR | CEF | DOX | CIP | GEN | ERY | CHL |
| --- | --- | --- | --- | --- | --- | --- | --- | --- | --- | --- |
| CC16101 | 32 | <0.06 | <0.06 | 512 | >512 | 128 | 32 | 256 | >512 | <0.06 |
| CC15071 | 4 | >512 | >512 | 32 | >512 | 64 | 4 | 256 | >512 | 32 |
| CC16091 | 16 | >512 | >512 | 512 | >512 | 128 | 32 | 32 | >512 | 256 |
| CC16082 | 8 | 32 | >512 | 128 | >512 | 128 | 16 | >512 | >512 | 32 |
| CC15042 | 8 | >512 | >512 | 128 | >512 | 128 | 16 | >512 | >512 | 32 |
| CC15031 | 16 | >512 | >512 | 32 | >512 | 128 | 16 | 256 | >512 | 256 |
| CC15091 | 16 | >512 | >512 | 32 | >512 | 64 | 4 | 256 | >512 | 16 |
| CC16092 | 32 | >512 | >512 | 512 | >512 | 256 | 32 | 256 | >512 | 128 |
| CC16071 | <0.06 | <0.06 | 64 | 2 | <0.06 | 16 | 0.125 | 0.5 | 128 | 16 |
| CC16051 | 128 | >512 | 512 | >512 | >512 | 256 | 64 | >512 | >512 | >512 |
| CC17011 | 16 | >512 | >512 | 128 | <0.06 | 128 | 16 | 0.125 | >512 | 32 |
| CC16012 | 0.5 | 32 | 128 | 4 | <0.06 | 2 | 0.125 | 0.125 | 128 | 256 |

ENR, Enrofloxacin; CLI, Clindamycin; SUL, Sulfamethoxazole; FOR, Florfenicol; CEF, Ceftriaxone; DOX, Doxycycline; CIP, Ciprofloxacin; GEN, Gentamicin; ERY, Erythromycin; CHL, Chloramphenicol677


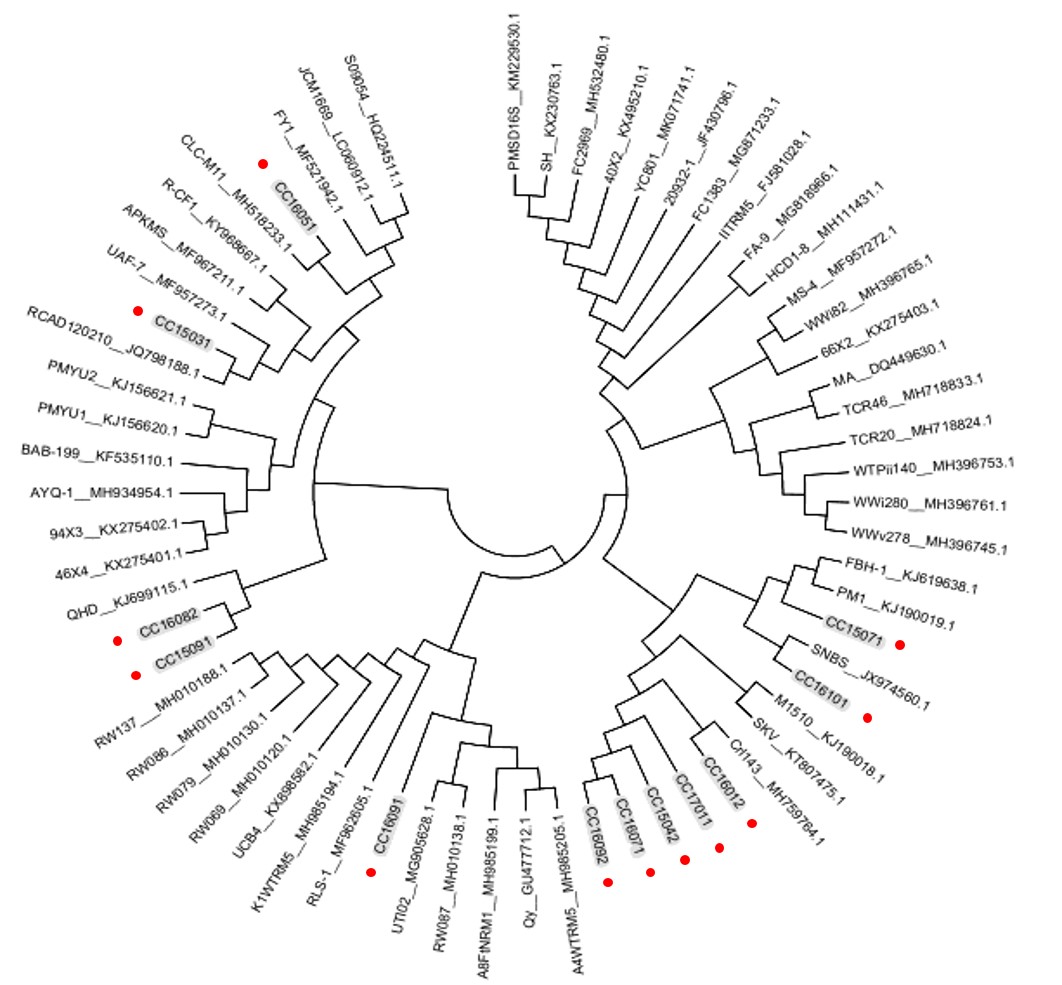


Supplement Figure S1. The phylogenetic tree based on 16S rRNA gene sequences of *P. mirabilis*.

Red represents 12 strains isolated from the diarrhea samples of dogs. Black represents 52 strains of *P. mirabilis*, and the sequence is included in GenBank.

***
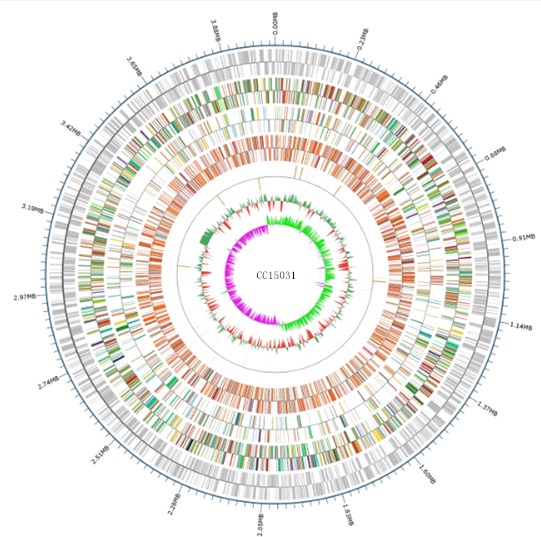
***

Supplement Figure S2. Relevant genomic features of CC15031 chromosome

Moving inward from the outermost circle, each ring of the circle contains information of a genome: rRNA/tRNA (red for tRNA and blue for rRNAs), reverse CDSs (colored according to the COGs categories), CDSs (colored according to the COGs categories), GC skew (yellow and blue for positive and negative values, respectively) and GC ratio (red and green for positive and negative values, respectively).

Supplement Table S3. Annotation of CC15031 based on VFDB

| Gene_id | Identity | | E_value | VFDB_internal_  id | VF_id | VF_name |
| --- | --- | --- | --- | --- | --- | --- |
| GM000027 | 42.7 | 4.40E-34 | | VFG000320 | VF0056 | LPS |
| GM000028 | 49.4 | 2.50E-63 | | VFG013315 | CVF494 | LOS |
| GM000029 | 51.9 | 5.70E-121 | | VFG013157 | CVF494 | LOS |
| GM000034 | 58 | 4.40E-94 | | VFG013152 | CVF494 | LOS |
| GM000035 | 60.9 | 6.50E-115 | | VFG013400 | CVF494 | LOS |
| GM000036 | 78.1 | 3.60E-141 | | VFG013409 | CVF494 | LOS |
| GM000049 | 66 | 6.00E-154 | | VFG007656 | CVF282 | Capsular polysaccharide |
| GM000050 | 63.1 | 6.30E-117 | | VFG019018 | CVF043 | O-antigen |
| GM000051 | 49.2 | 2.10E-70 | | VFG038088 | CVF775 | Capsule |
| GM000058 | 40.6 | 5.20E-36 | | VFG009866 | CVF333 | MprA/B |
| GM000120 | 82.4 | 1.20E-194 | | VFG046459 | CVF827 | EF-Tu |
| GM000162 | 40.8 | 3.80E-29 | | VFG045340 | VF0414 | RicA |
| GM000186 | 57.5 | 1.10E-106 | | VFG013510 | CVF494 | LOS |
| GM000187 | 65.6 | 9.40E-130 | | VFG023778 | CVF043 | O-antigen |
| GM000189 | 71.2 | 6.50E-153 | | VFG007640 | CVF282 | Capsular polysaccharide |
| GM000190 | 68.9 | 1.80E-164 | | VFG007635 | CVF282 | Capsular polysaccharide |
| GM000191 | 80.1 | 2.70E-169 | | VFG007659 | CVF282 | Capsular polysaccharide |
| GM000192 | 74.4 | 4.00E-126 | | VFG037938 | CVF775 | Capsule |
| GM000201 | 40.4 | 1.40E-60 | | VFG013634 | CVF506 | Heme biosynthesis |
| GM000202 | 44.7 | 3.30E-49 | | VFG013199 | CVF506 | Heme biosynthesis |
| GM000203 | 64.4 | 5.80E-107 | | VFG013198 | CVF506 | Heme biosynthesis |
| GM000252 | 43 | 1.20E-39 | | VFG010532 | CVF349 | Mip |
| GM000256 | 60.3 | 1.10E-149 | | VFG038900 | CVF792 | Hemolysin, HlyA |
| GM000282 | 74.3 | 4.20E-191 | | VFG013514 | CVF495 | Exopolysaccharide |
| GM000284 | 47.6 | 1.90E-31 | | VFG039487 | CVF803 | T4SS effectors |
| GM000291 | 76.4 | 5.30E-126 | | VFG043545 | AI331 | NlpI |
| GM000306 | 56.1 | 3.90E-50 | | VFG043619 | AI075 | type 1 fimbriae |
| GM000307 | 56.6 | 2.60E-69 | | VFG033257 | CVF426 | Type I fimbriae |
| GM000308 | 53.4 | 3.90E-278 | | VFG033294 | CVF426 | Type I fimbriae |
| GM000309 | 52.1 | 1.20E-43 | | VFG012305 | CVF426 | Type I fimbriae |
| GM000310 | 52.1 | 1.40E-46 | | VFG012313 | CVF426 | Type I fimbriae |
| GM000311 | 49 | 2.40E-76 | | VFG042718 | AI090 | F9 fimbriae |
| GM000326 | 43.4 | 2.00E-59 | | VFG047700 | CVF845 | Pyrimidine biosynthesis |
| GM000327 | 43.7 | 5.70E-68 | | VFG015903 | CVF546 | Phytotoxin phaseolotoxin |
| GM000392 | 49.4 | 1.40E-46 | | VFG013201 | CVF506 | Heme biosynthesis |
| GM000407 | 49.3 | 0.00E+00 | | VFG009597 | CVF318 | Nitrate reductase |
| GM000408 | 56.7 | 3.10E-166 | | VFG024081 | CVF318 | Nitrate reductase |
| GM000416 | 44.5 | 4.80E-77 | | VFG019760 | CVF518 | Type IV pili biosynthesis |
| GM000435 | 40.5 | 4.80E-63 | | VFG036990 | CVF759 | MtrCDE |
| GM000436 | 43.3 | 4.40E-244 | | VFG037008 | CVF759 | MtrCDE |
| GM000451 | 46.9 | 2.20E-89 | | VFG030679 | CVF651 | Trehalose-recycling ABC transporter |
| GM000454 | 49.5 | 5.10E-117 | | VFG043534 | AI309 | SP41/UgpB |
| GM000484 | 55.6 | 9.30E-152 | | VFG042736 | AI097 | type IV pili |
| GM000489 | 46.1 | 3.50E-70 | | VFG011729 | CVF393 | Capsule biosynthesis and transport |
| GM000499 | 40.5 | 1.30E-70 | | VFG014988 | CVF523 | Alginate regulation |
| GM000500 | 40 | 3.70E-73 | | VFG015769 | CVF523 | Alginate regulation |
| GM000519 | 61 | 6.60E-28 | | VFG006471 | CVF221 | Urease |
| GM000520 | 47.2 | 2.10E-24 | | VFG000269 | VF0050 | Urease |
| GM000521 | 62.6 | 2.70E-211 | | VFG006476 | CVF221 | Urease |
| GM000524 | 68.1 | 1.20E-58 | | VFG006495 | CVF221 | Urease |
| GM000526 | 40 | 1.50E-31 | | VFG011801 | CVF396 | LOS |
| GM000560 | 45.9 | 2.40E-71 | | VFG010515 | SS047 | Dot/Icm |
| GM000562 | 56.3 | 4.10E-200 | | VFG043573 | AI392 | MOMP |
| GM000574 | 47.6 | 2.00E-96 | | VFG047721 | CVF845 | Pyrimidine biosynthesis |
| GM000575 | 57.5 | 0.00E+00 | | VFG047710 | CVF845 | Pyrimidine biosynthesis |
| GM000606 | 40.3 | 2.80E-42 | | VFG009865 | CVF333 | MprA/B |
| GM000622 | 49.9 | 1.10E-143 | | VFG047605 | CVF840 | Cysteine acquisition |
| GM000623 | 41.1 | 7.70E-28 | | VFG022828 | CVF322 | AhpC |
| GM000666 | 64.4 | 4.20E-69 | | VFG000077 | VF0074 | ClpP |
| GM000682 | 47 | 5.90E-273 | | VFG036998 | CVF759 | MtrCDE |
| GM000683 | 42.6 | 5.90E-80 | | VFG036980 | CVF759 | MtrCDE |
| GM000733 | 45.6 | 4.30E-08 | | VFG042629 | AI072 | Proteus-like (MR/P) fimbriae, mannose resistant (biofilm formation) |
| GM000738 | 44.3 | 3.10E-61 | | VFG030684 | CVF651 | Trehalose-recycling ABC transporter |
| GM000744 | 53.1 | 8.80E-30 | | VFG008117 | CVF305 | Pantothenate synthesis |
| GM000745 | 48.1 | 1.20E-55 | | VFG009302 | CVF305 | Pantothenate synthesis |
| GM000754 | 70.1 | 9.90E-182 | | VFG013203 | CVF506 | Heme biosynthesis |
| GM000760 | 43 | 1.50E-180 | | VFG001887 | VF0260 | RelA |
| GM000764 | 59.9 | 7.20E-140 | | VFG005579 | CVF153 | Streptococcal enolase |
| GM000767 | 44.3 | 3.40E-52 | | VFG041913 | SS003 | T3SS |
| GM000777 | 98.8 | 1.20E-176 | | VFG044280 | IA034 | Proteobactin |
| GM000778 | 98.9 | 1.60E-142 | | VFG044281 | IA034 | Proteobactin |
| GM000779 | 99.6 | 1.40E-157 | | VFG044282 | IA034 | Proteobactin |
| GM000780 | 98.9 | 0.00E+00 | | VFG044283 | IA034 | Proteobactin |
| GM000781 | 99.1 | 0.00E+00 | | VFG044284 | IA034 | Proteobactin |
| GM000782 | 98.9 | 1.30E-275 | | VFG044285 | IA034 | Proteobactin |
| GM000783 | 99.4 | 1.50E-193 | | VFG044286 | IA034 | Proteobactin |
| GM000784 | 98.4 | 2.00E-226 | | VFG044287 | IA034 | Proteobactin |
| GM000785 | 99.8 | 6.60E-228 | | VFG044288 | IA034 | Proteobactin |
| GM000786 | 100 | 9.10E-213 | | VFG044289 | IA034 | Proteobactin |
| GM000787 | 100 | 1.60E-83 | | VFG044290 | IA034 | Proteobactin |
| GM000802 | 78.9 | 4.80E-74 | | VFG042621 | AI072 | Proteus-like (MR/P) fimbriae, mannose resistant (biofilm formation) |
| GM000803 | 66.1 | 0.00E+00 | | VFG042623 | AI072 | Proteus-like (MR/P) fimbriae, mannose resistant (biofilm formation) |
| GM000804 | 74.2 | 2.70E-110 | | VFG042624 | AI072 | Proteus-like (MR/P) fimbriae, mannose resistant (biofilm formation) |
| GM000805 | 47.3 | 1.60E-43 | | VFG042625 | AI072 | Proteus-like (MR/P) fimbriae, mannose resistant (biofilm formation) |
| GM000806 | 62.1 | 7.90E-49 | | VFG042626 | AI072 | Proteus-like (MR/P) fimbriae, mannose resistant (biofilm formation) |
| GM000807 | 55.5 | 7.80E-48 | | VFG042627 | AI072 | Proteus-like (MR/P) fimbriae, mannose resistant (biofilm formation) |
| GM000808 | 47.7 | 1.70E-65 | | VFG042628 | AI072 | Proteus-like (MR/P) fimbriae, mannose resistant (biofilm formation) |
| GM000809 | 64.2 | 1.10E-33 | | VFG042629 | AI072 | Proteus-like (MR/P) fimbriae, mannose resistant (biofilm formation) |
| GM000810 | 100 | 1.60E-108 | | VFG042620 | AI072 | Proteus-like (MR/P) fimbriae, mannose resistant (biofilm formation) |
| GM000811 | 100 | 9.20E-94 | | VFG042621 | AI072 | Proteus-like (MR/P) fimbriae, mannose resistant (biofilm formation) |
| GM000812 | 99.5 | 5.20E-103 | | VFG042622 | AI072 | Proteus-like (MR/P) fimbriae, mannose resistant (biofilm formation) |
| GM000813 | 100 | 0.00E+00 | | VFG042623 | AI072 | Proteus-like (MR/P) fimbriae, mannose resistant (biofilm formation) |
| GM000814 | 100 | 6.90E-143 | | VFG042624 | AI072 | Proteus-like (MR/P) fimbriae, mannose resistant (biofilm formation) |
| GM000815 | 100 | 8.10E-101 | | VFG042625 | AI072 | Proteus-like (MR/P) fimbriae, mannose resistant (biofilm formation) |
| GM000816 | 100 | 6.30E-89 | | VFG042626 | AI072 | Proteus-like (MR/P) fimbriae, mannose resistant (biofilm formation) |
| GM000817 | 100 | 5.20E-100 | | VFG042627 | AI072 | Proteus-like (MR/P) fimbriae, mannose resistant (biofilm formation) |
| GM000818 | 100 | 7.50E-159 | | VFG042628 | AI072 | Proteus-like (MR/P) fimbriae, mannose resistant (biofilm formation) |
| GM000819 | 100 | 8.50E-58 | | VFG042629 | AI072 | Proteus-like (MR/P) fimbriae, mannose resistant (biofilm formation) |
| GM000825 | 41.3 | 3.80E-88 | | VFG044152 | IA042 | HasA-type hemophore-mediated heme uptake system |
| GM000826 | 52.8 | 2.50E-164 | | VFG044153 | IA042 | HasA-type hemophore-mediated heme uptake system |
| GM000827 | 92.5 | 8.70E-278 | | VFG043807 | TX178 | ZapA |
| GM000840 | 43.1 | 1.90E-08 | | VFG042629 | AI072 | Proteus-like (MR/P) fimbriae, mannose resistant (biofilm formation) |
| GM000843 | 43.4 | 4.60E-191 | | VFG042585 | AI046 | Sfp fimbriae |
| GM000844 | 54.6 | 6.50E-68 | | VFG042654 | AI078 | Pix pilus |
| GM000864 | 42.6 | 2.70E-27 | | VFG002308 | VF0401 | Type IV pili |
| GM000878 | 68.7 | 1.40E-155 | | VFG013626 | CVF506 | Heme biosynthesis |
| GM000879 | 55.4 | 2.70E-57 | | VFG013268 | CVF494 | LOS |
| GM000883 | 44.2 | 1.10E-74 | | VFG042917 | AI117 | type IV pili |
| GM000887 | 49.6 | 7.10E-60 | | VFG045607 | VF0156 | Dot/Icm |
| GM000895 | 78.1 | 8.60E-85 | | VFG013418 | CVF494 | LOS |
| GM000909 | 49.4 | 3.90E-204 | | VFG044365 | IA012 | Chrysobactin |
| GM000923 | 83.6 | 3.70E-21 | | VFG010906 | CVF362 | Carbon storage regulator A |
| GM000926 | 77.8 | 2.70E-74 | | VFG018243 | CVF628 | Autoinducer-2 |
| GM000936 | 46 | 2.10E-79 | | VFG044185 | IA023 | Vulnibactin |
| GM000945 | 43.8 | 3.60E-183 | | VFG000079 | VF0072 | ClpC |
| GM000954 | 58.2 | 4.30E-176 | | VFG036965 | CVF758 | FarAB |
| GM000955 | 46.5 | 2.80E-90 | | VFG036944 | CVF758 | FarAB |
| GM000961 | 45.6 | 1.30E-192 | | VFG013643 | CVF506 | Heme biosynthesis |
| GM000964 | 40.6 | 9.00E-44 | | VFG030684 | CVF651 | Trehalose-recycling ABC transporter |
| GM001002 | 41.5 | 2.50E-40 | | VFG030314 | CVF649 | MymA operon |
| GM001155 | 94.1 | 2.70E-48 | | VFG042615 | AI071 | uroepithelial cell adhesin (UCA) |
| GM001156 | 99.2 | 2.60E-207 | | VFG042616 | AI071 | uroepithelial cell adhesin (UCA) |
| GM001157 | 99.1 | 0.00E+00 | | VFG042617 | AI071 | uroepithelial cell adhesin (UCA) |
| GM001158 | 98.2 | 2.40E-126 | | VFG042618 | AI071 | uroepithelial cell adhesin (UCA) |
| GM001159 | 95 | 2.30E-92 | | VFG042619 | AI071 | uroepithelial cell adhesin (UCA) |
| GM001165 | 86.4 | 9.00E-74 | | VFG000478 | VF0113 | Fur |
| GM001207 | 40.4 | 1.90E-14 | | VFG039074 | CVF799 | OmpA |
| GM001212 | 51.8 | 2.00E-87 | | VFG044185 | IA023 | Vulnibactin |
| GM001226 | 51 | 1.60E-89 | | VFG047511 | CVF838 | Biotin metabolism |
| GM001259 | 41.6 | 3.00E-45 | | VFG044378 | IA009 | thioquinolobactin |
| GM001312 | 40.4 | 7.40E-71 | | VFG000079 | VF0072 | ClpC |
| GM001339 | 67.8 | 3.40E-225 | | VFG013149 | CVF494 | LOS |
| GM001340 | 51.9 | 1.00E-93 | | VFG013246 | CVF494 | LOS |
| GM001343 | 70.9 | 3.20E-95 | | VFG038845 | VF0473 | Polar flagella |
| GM001354 | 52.3 | 9.60E-42 | | VFG035450 | CVF736 | ACE T6SS |
| GM001357 | 49.5 | 0.00E+00 | | VFG035495 | CVF736 | ACE T6SS |
| GM001358 | 44.3 | 1.30E-105 | | VFG035513 | CVF736 | ACE T6SS |
| GM001359 | 47.2 | 5.70E-41 | | VFG035543 | CVF736 | ACE T6SS |
| GM001361 | 64.3 | 0.00E+00 | | VFG035582 | CVF736 | ACE T6SS |
| GM001362 | 55.6 | 1.10E-74 | | VFG035601 | CVF736 | ACE T6SS |
| GM001363 | 46.4 | 2.50E-122 | | VFG038391 | SS194 | T6SS |
| GM001364 | 50 | 4.40E-46 | | VFG038389 | SS194 | T6SS |
| GM001365 | 41.8 | 1.90E-84 | | VFG038387 | SS194 | T6SS |
| GM001366 | 48.1 | 2.70E-89 | | VFG038385 | SS194 | T6SS |
| GM001367 | 40.8 | 1.90E-127 | | VFG038384 | CVF782 | T6SS |
| GM001368 | 43.4 | 5.00E-29 | | VFG038381 | SS194 | T6SS |
| GM001369 | 76.6 | 4.20E-229 | | VFG038380 | SS194 | T6SS |
| GM001370 | 65.6 | 2.50E-56 | | VFG038379 | CVF782 | T6SS |
| GM001372 | 90.1 | 5.00E-92 | | VFG038370 | VF0480 | T6SS |
| GM001401 | 54.1 | 9.90E-109 | | VFG043568 | AI384 | Hek |
| GM001420 | 69.1 | 1.00E-142 | | VFG043544 | AI330 | OmpA |
| GM001444 | 42.7 | 1.10E-65 | | VFG013174 | CVF494 | LOS |
| GM001481 | 44.2 | 3.50E-157 | | VFG007263 | CVF277 | Enterobactin receptors |
| GM001499 | 77.5 | 3.10E-103 | | VFG038840 | VF0473 | Polar flagella |
| GM001500 | 63.2 | 1.00E-20 | | VFG011430 | CVF383 | LPS |
| GM001501 | 41.4 | 5.60E-78 | | VFG009135 | CVF300 | FAS-II |
| GM001522 | 54 | 2.50E-149 | | VFG018397 | CVF010 | PhoPQ |
| GM001523 | 69.5 | 2.50E-91 | | VFG004061 | CVF010 | PhoPQ |
| GM001574 | 61.2 | 6.00E-19 | | VFG042615 | AI071 | uroepithelial cell adhesin (UCA) |
| GM001591 | 61.5 | 3.70E-18 | | VFG042615 | AI071 | uroepithelial cell adhesin (UCA) |
| GM001635 | 50.7 | 1.80E-75 | | VFG012586 | CVF459 | Iron/managanease transport |
| GM001636 | 66.8 | 2.10E-106 | | VFG012585 | CVF459 | Iron/managanease transport |
| GM001637 | 62 | 1.60E-90 | | VFG034214 | CVF459 | Iron/managanease transport |
| GM001638 | 61.9 | 4.30E-107 | | VFG012575 | CVF459 | Iron/managanease transport |
| GM001644 | 50.5 | 4.40E-83 | | VFG042718 | AI090 | F9 fimbriae |
| GM001652 | 40.4 | 2.10E-57 | | VFG044237 | IA059 | direct heme uptake system |
| GM001654 | 41.2 | 1.00E-81 | | VFG011156 | CVF380 | LPS |
| GM001664 | 47 | 1.10E-105 | | VFG012874 | CVF465 | Mxi-Spa TTSS effectors controlled by MxiE |
| GM001675 | 48.6 | 3.70E-55 | | VFG042564 | AI042 | F18 fimbriae |
| GM001688 | 51.4 | 1.20E-115 | | VFG013196 | CVF506 | Heme biosynthesis |
| GM001692 | 80.6 | 2.50E-133 | | VFG013466 | CVF494 | LOS |
| GM001717 | 60.5 | 2.30E-82 | | VFG039536 | CVF803 | T4SS effectors |
| GM001721 | 90.1 | 5.00E-92 | | VFG038370 | VF0480 | T6SS |
| GM001723 | 41.5 | 1.10E-72 | | VFG035856 | CVF736 | ACE T6SS |
| GM001736 | 58.6 | 3.20E-108 | | VFG013087 | CVF484 | MsbB2 |
| GM001762 | 41.5 | 6.50E-79 | | VFG025879 | CVF643 | Flagella |
| GM001810 | 42 | 2.90E-39 | | VFG009864 | CVF333 | MprA/B |
| GM001877 | 47.1 | 1.20E-31 | | VFG035450 | CVF736 | ACE T6SS |
| GM001914 | 54.2 | 6.00E-76 | | VFG045346 | VF0513 | IlpA |
| GM001928 | 60.5 | 6.60E-60 | | VFG000463 | VF0109 | SodCI |
| GM001938 | 40.9 | 8.10E-61 | | VFG026700 | CVF315 | Mycobactin |
| GM001954 | 45.5 | 7.80E-40 | | VFG003498 | CVF037 | Ail |
| GM001965 | 42 | 2.30E-60 | | VFG043148 | AI142 | lateral flagella |
| GM001973 | 40.4 | 1.00E-56 | | VFG001214 | VF0082 | Type IV pili |
| GM001995 | 66.5 | 2.30E-77 | | VFG001867 | VF0169 | SodB |
| GM002020 | 55 | 2.30E-96 | | VFG044185 | IA023 | vulnibactin |
| GM002021 | 47.5 | 2.50E-08 | | VFG044241 | IA059 | direct heme uptake system |
| GM002023 | 100 | 0.00E+00 | | VFG044291 | IA060 | direct heme uptake system |
| GM002024 | 100 | 1.60E-209 | | VFG044292 | IA060 | direct heme uptake system |
| GM002025 | 100 | 1.50E-140 | | VFG044293 | IA060 | direct heme uptake system |
| GM002026 | 100 | 1.50E-185 | | VFG044294 | IA060 | direct heme uptake system |
| GM002027 | 100 | 3.60E-150 | | VFG044295 | IA060 | direct heme uptake system |
| GM002052 | 42.8 | 1.20E-62 | | VFG013197 | CVF506 | Heme biosynthesis |
| GM002064 | 44 | 1.10E-219 | | VFG012302 | CVF426 | Type I fimbriae |
| GM002065 | 50.2 | 3.80E-63 | | VFG042681 | AI083 | type 1 fimbriae |
| GM002067 | 48.1 | 4.60E-43 | | VFG021131 | CVF003 | Fim |
| GM002069 | 47.3 | 1.90E-34 | | VFG016532 | CVF591 | Capsule |
| GM002086 | 49.1 | 8.70E-233 | | VFG006717 | VF0444 | Lap |
| GM002089 | 49.9 | 6.70E-93 | | VFG016390 | CVF567 | Polysaccharide capsule |
| GM002090 | 70 | 7.80E-117 | | VFG013348 | CVF494 | LOS |
| GM002107 | 49.7 | 1.90E-87 | | VFG005359 | CVF123 | Streptococcal plasmin receptor/GAPDH |
| GM002125 | 41.6 | 6.10E-27 | | VFG019944 | CVF529 | GacS/GacA two-component system |
| GM002141 | 44.9 | 2.70E-36 | | VFG013192 | CVF501 | Haemophilus iron transport locus |
| GM002155 | 42.3 | 3.40E-53 | | VFG044099 | IA049 | direct heme uptake system |
| GM002179 | 44.3 | 1.10E-71 | | VFG047558 | CVF839 | Purine |
| GM002199 | 45.7 | 1.80E-35 | | VFG011225 | CVF381 | LPS-modifying enzyme |
| GM002226 | 67.3 | 3.60E-61 | | VFG002318 | VF0394 | Flagella |
| GM002227 | 83.1 | 2.40E-108 | | VFG002319 | VF0394 | Flagella |
| GM002228 | 57.2 | 8.60E-102 | | VFG011232 | CVF382 | Flagella |
| GM002229 | 60.1 | 5.20E-107 | | VFG011232 | CVF382 | Flagella |
| GM002230 | 48.1 | 1.70E-113 | | VFG002324 | VF0394 | Flagella |
| GM002231 | 59.2 | 5.10E-36 | | VFG043053 | AI139 | peritrichous flagella |
| GM002232 | 43 | 1.50E-20 | | VFG002326 | VF0394 | Flagella |
| GM002238 | 58.3 | 1.50E-28 | | VFG003147 | CVF039 | Flagella (cluster I) |
| GM002239 | 59.4 | 1.80E-191 | | VFG023635 | CVF039 | Flagella (cluster I) |
| GM002240 | 81.7 | 2.00E-150 | | VFG002329 | VF0394 | Flagella |
| GM002241 | 42.9 | 2.40E-52 | | VFG002330 | VF0394 | Flagella |
| GM002242 | 79.6 | 7.50E-204 | | VFG023641 | CVF039 | Flagella (cluster I) |
| GM002243 | 62.1 | 1.90E-47 | | VFG002332 | VF0394 | Flagella |
| GM002245 | 57.1 | 6.80E-43 | | VFG023647 | CVF039 | Flagella (cluster I) |
| GM002246 | 79 | 2.20E-155 | | VFG002335 | VF0394 | Flagella |
| GM002247 | 67.9 | 9.50E-46 | | VFG043064 | AI139 | peritrichous flagella |
| GM002248 | 51.9 | 7.20E-23 | | VFG043065 | AI139 | peritrichous flagella |
| GM002249 | 78.3 | 2.90E-104 | | VFG023655 | CVF039 | Flagella (cluster I) |
| GM002250 | 77.5 | 1.40E-34 | | VFG002339 | VF0394 | Flagella |
| GM002251 | 61.5 | 2.70E-89 | | VFG002669 | CVF039 | Flagella (cluster I) |
| GM002253 | 46.4 | 6.30E-71 | | VFG002670 | CVF039 | Flagella (cluster I) |
| GM002254 | 50.9 | 1.90E-161 | | VFG002671 | CVF039 | Flagella (cluster I) |
| GM002255 | 54.8 | 4.40E-89 | | VFG002343 | VF0394 | Flagella |
| GM002256 | 76.5 | 1.70E-153 | | VFG043029 | AI139 | peritrichous flagella |
| GM002257 | 78.1 | 2.10E-96 | | VFG002345 | VF0394 | Flagella |
| GM002258 | 83.5 | 1.10E-122 | | VFG003433 | CVF039 | Flagella (cluster I) |
| GM002259 | 62.9 | 1.20E-86 | | VFG023672 | CVF039 | Flagella (cluster I) |
| GM002260 | 53 | 1.20E-123 | | VFG002677 | CVF039 | Flagella (cluster I) |
| GM002261 | 47.6 | 2.90E-19 | | VFG011281 | CVF382 | Flagella |
| GM002262 | 77.6 | 3.80E-55 | | VFG002679 | CVF039 | Flagella (cluster I) |
| GM002263 | 65 | 1.90E-46 | | VFG002351 | VF0394 | Flagella |
| GM002265 | 51.5 | 5.90E-21 | | VFG023684 | CVF039 | Flagella (cluster I) |
| GM002266 | 43.4 | 7.30E-28 | | VFG002683 | CVF039 | Flagella (cluster I) |
| GM002270 | 78 | 4.4e-312 | | VFG023690 | CVF039 | Flagella (cluster I) |
| GM002271 | 63.8 | 1.90E-139 | | VFG017341 | CVF039 | Flagella (cluster I) |
| GM002273 | 74.1 | 1.60E-82 | | VFG043205 | AI145 | peritrichous flagella |
| GM002274 | 83.7 | 2.50E-56 | | VFG043206 | AI145 | peritrichous flagella |
| GM002275 | 76.6 | 1.90E-151 | | VFG043038 | AI139 | peritrichous flagella |
| GM002276 | 63.8 | 9.40E-99 | | VFG043208 | AI145 | peritrichous flagella |
| GM002277 | 45.7 | 3.10E-116 | | VFG043209 | AI145 | peritrichous flagella |
| GM002278 | 55.6 | 2.00E-158 | | VFG043209 | AI145 | peritrichous flagella |
| GM002279 | 81 | 2.90E-65 | | VFG043210 | AI145 | peritrichous flagella |
| GM002280 | 65.3 | 7.50E-246 | | VFG043211 | AI145 | peritrichous flagella |
| GM002281 | 57.5 | 9.10E-101 | | VFG043212 | AI145 | peritrichous flagella |
| GM002282 | 71 | 4.40E-120 | | VFG043213 | AI145 | peritrichous flagella |
| GM002283 | 82.8 | 6.00E-86 | | VFG002687 | CVF039 | Flagella (cluster I) |
| GM002284 | 81 | 1.60E-46 | | VFG002359 | VF0394 | Flagella |
| GM002286 | 62.4 | 2.40E-73 | | VFG004044 | CVF005 | Mg2+ transport |
| GM002298 | 56.3 | 1.80E-95 | | VFG013438 | CVF494 | LOS |
| GM002354 | 68.8 | 4.80E-196 | | VFG037041 | CVF760 | Catalase |
| GM002429 | 52.4 | 3.60E-10 | | VFG042629 | AI072 | Proteus-like (MR/P) fimbriae, mannose resistant (biofilm formation) |
| GM002445 | 45.1 | 6.00E-55 | | VFG013192 | CVF501 | Haemophilus iron transport locus |
| GM002463 | 46.6 | 3.50E-27 | | VFG031464 | CVF660 | Nucleoside diphosphate kinase |
| GM002487 | 44.7 | 2.50E-95 | | VFG043345 | AI149 | polar flagella |
| GM002492 | 100 | 1.10E-97 | | VFG042610 | AI070 | PMF pili |
| GM002493 | 99.6 | 0.00E+00 | | VFG042611 | AI070 | PMF pili |
| GM002494 | 99.6 | 6.30E-144 | | VFG042612 | AI070 | PMF pili |
| GM002495 | 99.7 | 1.10E-210 | | VFG042613 | AI070 | PMF pili |
| GM002496 | 99.5 | 7.60E-99 | | VFG042614 | AI070 | PMF pili |
| GM002510 | 65.6 | 1.90E-68 | | VFG000121 | VF0091 | Alginate |
| GM002572 | 56.8 | 4.80E-231 | | VFG012633 | CVF461 | Iron-regulated element |
| GM002578 | 60.3 | 4.40E-110 | | VFG013287 | CVF494 | LOS |
| GM002589 | 51.2 | 1.20E-71 | | VFG038332 | VF0479 | T3SS |
| GM002616 | 48.7 | 1.10E-187 | | VFG007023 | CVF263 | RTX toxin |
| GM002617 | 46.5 | 4.80E-110 | | VFG007030 | CVF263 | RTX toxin |
| GM002618 | 49.3 | 1.20E-189 | | VFG038918 | CVF795 | The repeat in toxin (RTX) |
| GM002632 | 53.9 | 0.00E+00 | | VFG007017 | CVF263 | RTX toxin |
| GM002639 | 47.8 | 9.30E-31 | | VFG045955 | CVF825 | Hemorrhagic E.coli pilus (HCP) |
| GM002640 | 48.9 | 6.90E-91 | | VFG042799 | AI102 | Hemorrhagic Coli pili (HCP) |
| GM002646 | 99.3 | 0.00E+00 | | VFG042330 | SS132 | HpmA-HpmB |
| GM002647 | 99.6 | 0.00E+00 | | VFG042331 | SS132 | HpmA-HpmB |
| GM002651 | 73.7 | 6.50E-135 | | VFG013417 | CVF494 | LOS |
| GM002673 | 48.5 | 7.70E-44 | | VFG009376 | CVF309 | Leucine synthesis |
| GM002706 | 98.8 | 1.30E-138 | | VFG042332 | SS172 | AipA |
| GM002774 | 50.2 | 1.00E-63 | | VFG013326 | CVF494 | LOS |
| GM002787 | 41.2 | 1.30E-133 | | VFG031407 | CVF658 | Copper exporter |
| GM002796 | 41.7 | 6.80E-70 | | VFG002689 | CVF043 | O-antigen |
| GM002797 | 49.4 | 4.00E-87 | | VFG013617 | CVF506 | Heme biosynthesis |
| GM002822 | 43.8 | 1.30E-08 | | VFG042629 | AI072 | Proteus-like (MR/P) fimbriae, mannose resistant (biofilm formation) |
| GM002826 | 48.2 | 2.50E-128 | | VFG021660 | CVF027 | Sti |
| GM002827 | 42.8 | 3.90E-41 | | VFG012335 | VF0222 | S fimbriae |
| GM002832 | 40.7 | 1.10E-28 | | VFG033094 | CVF425 | P fimbriae |
| GM002835 | 63.6 | 1.60E-88 | | VFG042586 | AI046 | Sfp fimbriae |
| GM002836 | 58.3 | 4.00E-304 | | VFG042585 | AI046 | Sfp fimbriae |
| GM002838 | 52 | 6.10E-40 | | VFG042651 | AI078 | Pix pilus |
| GM002845 | 79.9 | 1.50E-145 | | VFG000477 | VF0112 | RpoS |
| GM002853 | 49.7 | 1.20E-47 | | VFG015885 | CVF546 | Phytotoxin phaseolotoxin |
| GM002869 | 49 | 3.10E-39 | | VFG002552 | VF0436 | Capsule I |
| GM002872 | 63.5 | 6.00E-100 | | VFG045346 | VF0513 | IlpA |
| GM002883 | 63 | 3.20E-131 | | VFG013386 | CVF494 | LOS |
| GM002884 | 68.2 | 1.70E-102 | | VFG013167 | CVF494 | LOS |
| GM002885 | 47.2 | 1.70E-32 | | VFG011402 | CVF383 | LPS |
| GM002886 | 68.6 | 3.40E-132 | | VFG013165 | CVF494 | LOS |
| GM002889 | 43.9 | 6.20E-102 | | VFG015009 | CVF523 | Alginate regulation |
| GM002891 | 44.6 | 3.60E-53 | | VFG045688 | CVF618 | Capsule |
| GM002934 | 40 | 1.10E-33 | | VFG030679 | CVF651 | Trehalose-recycling ABC transporter |
| GM002950 | 99.4 | 0.00E+00 | | VFG042333 | SS112 | Proteus toxic agglutinin (Pta) |
| GM002958 | 65.5 | 1.20E-164 | | VFG044140 | IA044 | HasA-type hemophore-mediated heme uptake system |
| GM002968 | 70.4 | 2.70E-191 | | VFG013435 | CVF494 | LOS |
| GM002978 | 40.5 | 4.70E-36 | | VFG015510 | CVF536 | Phenazines biosynthesis |
| GM002982 | 53.2 | 3.70E-81 | | VFG009718 | CVF325 | Sigma A |
| GM002991 | 42.3 | 6.40E-71 | | VFG043040 | AI139 | peritrichous flagella |
| GM002992 | 40.4 | 1.70E-63 | | VFG043040 | AI139 | peritrichous flagella |
| GM003096 | 98.6 | 4.50E-78 | | VFG038025 | CVF775 | Capsule |
| GM003097 | 100 | 7.40E-90 | | VFG038010 | CVF775 | Capsule |
| GM003245 | 74.9 | 5.20E-228 | | VFG010484 | CVF347 | Hsp60 |
| GM003270 | 44.5 | 1.30E-46 | | VFG043577 | AI396 | M. catarrhalis adherence protein (McaP) |
| GM003284 | 40.8 | 1.00E-56 | | VFG012628 | CVF460 | Hemin uptake |
| GM003292 | 50 | 1.70E-44 | | VFG041856 | SS016 | Cpi-1a + Cpi-1 (SPI-1 like) |
| GM003293 | 43.2 | 1.70E-83 | | VFG041463 | SS005 | PAI-2 encoded |
| GM003294 | 42 | 8.30E-51 | | VFG003517 | SS018 | Ysa T3SS |
| GM003295 | 63 | 2.00E-25 | | VFG002454 | VF0428 | Bsa T3SS |
| GM003296 | 59.6 | 3.60E-71 | | VFG002455 | VF0428 | Bsa T3SS |
| GM003300 | 52.8 | 3.40E-126 | | VFG003523 | SS018 | Ysa T3SS |
| GM003302 | 53.4 | 2.80E-202 | | VFG003525 | SS018 | Ysa T3SS |
| GM003304 | 40.9 | 4.90E-104 | | VFG020107 | CVF462 | Mxi-Spa TTSA (type III secretion apparatus) |
| GM003308 | 47.9 | 1.20E-11 | | VFG012643 | CVF462 | Mxi-Spa TTSA (type III secretion apparatus) |
| GM003309 | 41.3 | 1.20E-07 | | VFG025179 | CVF641 | Bsa T3SS |
| GM003310 | 41.5 | 2.40E-39 | | VFG018554 | CVF031 | TTSS (SPI-1 encode) |
| GM003326 | 42.4 | 2.00E-77 | | VFG006720 | CVF228 | Listeria adhesion protein |
| GM003328 | 41.5 | 1.30E-101 | | VFG031960 | CVF228 | Listeria adhesion protein |
| GM003337 | 41.6 | 4.10E-31 | | VFG018389 | CVF003 | Fim |
| GM003338 | 44.1 | 1.80E-49 | | VFG042681 | AI083 | type 1 fimbriae |
| GM003339 | 47 | 2.20E-225 | | VFG033271 | CVF426 | Type I fimbriae |
| GM003340 | 40.7 | 3.10E-25 | | VFG043615 | AI075 | type 1 fimbriae |
| GM003346 | 46.2 | 9.50E-64 | | VFG030696 | CVF651 | Trehalose-recycling ABC transporter |
| GM003364 | 77.6 | 7.00E-257 | | VFG013531 | CVF495 | Exopolysaccharide |
| GM003373 | 61.3 | 1.40E-146 | | VFG001381 | VF0253 | Isocitrate lyase |
| GM003384 | 42.7 | 8.80E-16 | | VFG043551 | AI354 | histone-like protein (Hlp)/laminin-binding protein (LBP) |
| GM003387 | 78 | 2.50E-162 | | VFG013200 | CVF506 | Heme biosynthesis |
| GM003392 | 41.7 | 1.20E-46 | | VFG044378 | IA009 | thioquinolobactin |
| GM003405 | 82.2 | 3.60E-194 | | VFG046459 | CVF827 | EF-Tu |
| GM003421 | 48.9 | 1.40E-82 | | VFG025884 | CVF643 | Flagella |
| GM003422 | 40 | 1.90E-92 | | VFG043209 | AI145 | peritrichous flagella |
| GM003425 | 49.1 | 8.20E-41 | | VFG035450 | CVF736 | ACE T6SS |
| GM003426 | 45.2 | 2.90E-63 | | VFG043209 | AI145 | peritrichous flagella |
| GM003434 | 65.8 | 1.10E-74 | | VFG042734 | AI097 | type IV pili |
| GM003457 | 41.2 | 1.20E-28 | | VFG016532 | CVF591 | Capsule |
| GM003458 | 41.9 | 4.90E-24 | | VFG016532 | CVF591 | Capsule |
| GM003478 | 40.3 | 1.50E-142 | | VFG009926 | CVF335 | (p)ppGpp synthesis and hydrolysis |
| GM003491 | 49.5 | 1.00E-131 | | VFG009407 | CVF311 | Glutamine synthesis |
| GM003493 | 42.5 | 7.50E-82 | | VFG042915 | AI117 | type IV pili |
| GM003494 | 68.5 | 2.90E-187 | | VFG013205 | CVF506 | Heme biosynthesis |
| GM003500 | 40.9 | 1.60E-43 | | VFG038219 | VF0463 | BfmRS |
| GM003529 | 55.8 | 5.00E-07 | | VFG045722 | CVF359 | Ferrous iron transport |
| GM003530 | 46.9 | 1.80E-202 | | VFG047394 | CVF836 | Ferrous iron-transport system |
| GM003567 | 45.8 | 2.20E-79 | | VFG044221 | IA028 | petrobactin |
| GM003569 | 56.4 | 4.70E-75 | | VFG044219 | IA028 | petrobactin |
| GM003601 | 42.7 | 3.10E-49 | | VFG034435 | CVF625 | E. coli common pilus (ECP) |
| GM003602 | 52.6 | 4.20E-45 | | VFG034418 | CVF625 | E. coli common pilus (ECP) |
| GM003621 | 45.9 | 3.90E-67 | | VFG045346 | VF0513 | IlpA |
| GM003627 | 41.5 | 2.90E-57 | | VFG013118 | CVF486 | Type IV pili |
| GM003632 | 65.8 | 2.00E-77 | | VFG046612 | CVF833 | Capsule |
| GM003638 | 45.9 | 1.20E-45 | | VFG001867 | VF0169 | SodB |
| GM003670 | 52 | 7.20E-130 | | VFG047249 | CVF834 | LPS |
| GM003688 | 44.5 | 9.10E-56 | | VFG013192 | CVF501 | Haemophilus iron transport locus |
| GM003718 | 61.5 | 8.90E-103 | | VFG013348 | CVF494 | LOS |


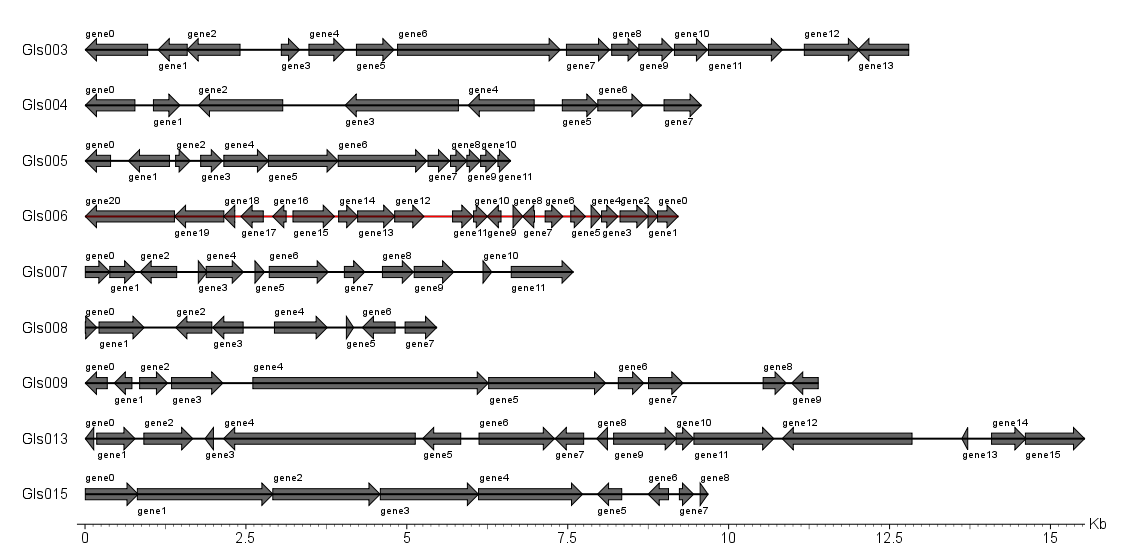
Supplement Figure S3. Statistical Map of Gene Distribution in Gene Island.
